# Biology, Population Fluctuation, and Foliar Consumption Rate of *Durrantia arcanella* Busk, 1912 (Lepidoptera: Depressariidae), a Defoliator of Oil Palm in the Colombian Caribbean

**DOI:** 10.3390/insects14120900

**Published:** 2023-11-21

**Authors:** German E. Tejeda-Rico, Carlos E. Barrios-Trilleras, Roberto J. Diaz-Castro, Leidy V. Florián-Martínez, Leidy J. Contreras-Arias, José Luis Padilla-Agudelo, Anuar Morales-Rodríguez

**Affiliations:** Colombian Oil Palm Research Centre, Cenipalma, Bogotá 252171, Colombia; gtejeda@cenipalma.org (G.E.T.-R.); robertojdiaz94@gmail.com (R.J.D.-C.); ljcontreras@cenipalma.org (L.J.C.-A.); jlpadilla@cenipalma.org (J.L.P.-A.);

**Keywords:** foliar consumption, recurring pest, natural enemy, climatic variables

## Abstract

**Simple Summary:**

Colombia currently has 595,722 oil palm-cultivated hectares, but production is declining due to phytophagous insects feeding mainly on the leaves; one of them, *Durrantia arcanella*, is a recurring pest in the northern palm zone of Colombia, for which we do not have all the essential information. Therefore, it was proposed to determine its biology, foliar consumption rate, population fluctuation, and relationship with climatic variables and to identify its main natural enemies in the department of Cesar. The life cycle under laboratory conditions, including adult longevity, was 48.0 ± 10.1 days, the egg stage lasted 8.0 ± 0.7 days, the larva stage lasted 24.2 ± 6.2 days, the pre-pupa stage lasted 1.5 ± 0.5 days, the pupa stage lasted 7.1 ± 0.9 and the adult had a longevity of 7.2 ± 2.0 days. At the end of the larval period, it was determined that they individually consumed 8.2 ± 5.3 cm^2^ of leaflets. Correlation was found between *D*. *arcanella* population dynamics and climatic factors such as temperature and relative humidity, likewise with natural enemies.

**Abstract:**

*Durrantia arcanella* is a recurring pest insect of oil palm in Colombia. Because the biology and ecology of *D. arcanella* are unknown, it was proposed to determine the life cycle and foliar consumption under laboratory conditions. Furthermore, through sequential sampling for two and a half years, its population fluctuation and natural enemies were determined in Agustín Codazzi and El Copey (Cesar, Colombia). Also, temperature, precipitation, and relative humidity were registered. The life cycle of *D. arcanella* lasted 48.0 ± 10.1 days, the egg 8.0 ± 0.7 days, larva 24.2 ± 6.2 days, pre-pupa 1.5 ± 0.5 days, pupa 7.1 ± 0.9 days, and adult 7.2 ± 2.0 days. The larvae consumed 8.2 ± 5.3 cm^2^ of leaflets. Correlations were found between the population fluctuation in *D. arcanella* and the temperature in El Copey (ρ = −0.45; *p* < 0.0043), relative humidity in Codazzi (ρ = 0.33; *p* < 0.034), and with the natural control in both locations ((ρ = 0, 61; *p* < 0.000044) and (ρ = 0.42; *p* < 0.006)). These results suggest monitoring the pest populations in the second semester of the year and show the importance of promoting native natural enemies.

## 1. Introduction

Oil palm cultivation is a vast global industry worth over USD 50 billion annually. Colombia currently has 595,722 cultivated hectares, is the fourth in world production, and is the first palm oil producer in the American continent [1,2].

The industrialization and planting of large areas of oil palm have transformed not only the lives of many people and the profits of companies dedicated to palm growing in tropical America but have also created profound changes in the ecosystem and, in turn, in the ecology of various phytophagous insects, which have been favored and adapted to a homogeneous and permanent environment where they maintain a balance of their populations at the expense of cultivation [3,4,5]. These insects can attack any plant tissue of the oil palm; however, the leaves are the primary food source for many arthropods. Most correspond mainly to defoliating insects of the order Lepidoptera, which usually cause the most significant economic losses in crops, decreasing production and increasing costs [3,6].

In oil palm plantations on the Caribbean coast of Colombia, defoliating insects are one of the main entomological problems to consider when implementing an Integrated Pest Management (IPM) program, one of them being the oil palm weaver worm, initially identified as *Durrantia* aff. *arcanella* (Busk, 1912), a moth of the Depressariidae family, microlepidoptera, with more than 3000 species distributed worldwide [3,7,8]. *Durrantia arcanella* has been registered to affect oil palm in countries such as Venezuela, Mexico, Honduras, Costa Rica, Panama, and Ecuador; the larvae cause direct damage by completely ingesting the leaf blade and indirect damage by generating a gateway to the complex of phytopathogenic fungi that cause the disease known as leaf blight or Pestalotiopsis [3,9,10,11].

When selecting an effective management strategy, some aspects are essential, such as correctly identifying the pest insect, understanding its biology and habits, the seasonal fluctuation in the population, its most damaging life stages, its foliar consumption rate, and its economic significance. These will allow for the pest insect’s impact on the crop to be measured and successful IPM programs to be developed [12,13,14,15]. The life cycle and habits of *D*. *arcanella* have been studied by [9,11]; however, updated and complete information on their biology and ecology in an environment of climate change is necessary [16], as well as the dynamics of their populations, the quantification of damage, and its natural enemies. Therefore, this research was carried out to corroborate the taxonomic identity of *D. arcanella* and determine the life cycle, population dynamics, foliar consumption rate, and natural enemies of *D. arcanella* in oil palm lots in the northern palm zone of Colombia.

## 2. Materials and Methods

**Molecular identification and phylogenetic analysis.** The genetic material was extracted from pupae (Copey_ Colombia 3), larvae (Copey_ Colombia 2), and adults (Copey_ Colombia 1) of *Durriantia* sp. using the NucleoSpin Plant II commercial kit following the supplier’s instructions. The extracted DNA was stored at −20 °C until further use. The amplified gene region corresponded to subunit 1 of cytochrome c oxidase (COI) with the primers LCO-1490 (5′-GGTCAACAAATCATAAAGATATTGG) and HCO-2198 (5′-TAAACTTCAGGGTGACCAAAAATCA) [17,18,19]. Reactions were incubated in a T3 thermocycler (Biometra, Gottingen, Germany) with a PCR reaction volume of 25 µL in each one, containing 12.5 of the GoTaq^®^ Green Master Mix kit (Promega, Madison, WI, USA), 0.5 µL of each of the primers (10 µM), 2 µL of DNA (200 ng/µL), and 9.5 µL of nuclease-free water. The reaction conditions were initial denaturation at 95 °C (5 min), 35 cycles of denaturation at 94 °C for 1 min, annealing at 55 °C for 30 s, and extension at 72 °C for 1 min, with a final extension at 72 °C for 10 min. After electrophoresis, the bands of the expected size were cut (fragment approx. 600 bp) and purified using the QIAquick Gel Extraction kit (Qiagen, Venlo, The Netherlands) and sent for sequencing at the National University of Colombia.

The sequences obtained with each primer were edited using the BioEdit 7.0.5.3 program [20], and the consensus sequence was constructed with the CAP contig assembly accessory for each isolate. Once the sequences were obtained, their identity was confirmed by comparing them with the GenBank (http://www.ncbi.nlm.nih.gov/BLAST) and Boldsystem (https://www.boldsystems.org/) databases accessed on 13 October 2023. According to the literature reported for phylogenetic analysis, the sequences generated in this study were complemented with additional sequences obtained from other related strains of *Durrantia* sp. Subsequently, sequence alignment was performed with the MUSCLE algorithm included in the MEGAX program [21]. The statistical method was the Maximum Likelihood method, and evolutionary distances were calculated using the Tamura–Nei method. This analysis involved 15 nucleotide sequences. Node support was determined using the bootstrap method with 1000 repetitions. *Carystus lota* Hewitson, 1877 (Lepidoptera: Hesperiidae) was used as an external group of the phylogenetic tree.

**Biology of *Durrantia arcanella*.** Larvae and pupae were collected during the year 2022 in the Palmeras de la Costa plantation, located in the municipality of El Copey (Cesar, Colombia) at the geographical coordinates 10°06′03.2″ N 74°00′35.0″ W. The individuals were transferred to the plantation laboratory in plastic jars; once there, the pupae were deposited in Petri dishes until the emergence of the adults, and the larvae were reared separately and fed inside a Petri dish containing a piece (8 × 5 cm) of leaflet taken from leaf 9 of the oil palm cultivar Compacta × Ghana, which was placed underneath a piece of absorbent paper moistened with sterile water to hydrate it, the paper and leaflet were replaced every three days until all larvae reached the pupal stage and adults emerged. The adults were transferred to 20 × 15 × 15 cm plastic containers with Kraft paper inside to allow oviposition and a piece of cotton moistened with a mixture of water and honey in a 1:1 ratio. The container was covered with a muslin cloth to prevent the escape of *D. arcanella* adults. The time from the laying of the eggs to the larvae hatching was recorded; these were reared separately in Petri dishes with a piece of leaflet and hydrated absorbent paper. The different larval instars’ development, morphological changes, and duration were observed and recorded daily. Subsequently, the duration of the pupae was determined, and, finally, the longevity of the adults was recorded.

**Population fluctuation in *D*. *arcanella*.** The study was carried out in two oil palm lots: The first lot of 31 ha planted in 2006 with *Elaeis guineensis* cultivar Compacta × Ghana, was located on Palmeras de la Costa plantation (El Copey, Cesar) at the geographic coordinates 10°06′38.4″ N 74°01′39.9″ W. The second lot, a 25.8 ha plot planted in 2008 with *E. guineensis* cultivar Deli × Avross, was located on Palmas Sicarare plantation (Agustín Codazzi, Cesar) at the geographic coordinates 09°55′28″ N 73°14′10″ W. Every 20 days, from December 2020 to June 2023, 5 × 5 sampling was carried out (every five palms every five lines until the entire lot was covered). On leaf 17 of each selected palm, the number of alive larvae, pupae, and those affected by natural enemies were recorded. Parasitized and individuals with signs of infection by entomopathogens were collected. The parasitoids were identified at the Fundación Instituto Entoma (Bogotá, D.C., Colombia), and the strains were isolated and identified in the laboratory of entomopathogenic micro-organisms—MEAPA of Cenipalma. Precipitation, temperature, and relative humidity were recorded during the sampling in both lots with Vantage Pro2™ weather stations (Davis Instruments, Hayward, CA, USA). Finally, Spearman correlations were performed between population fluctuation, climatic factors, and biological control exerted by natural enemies using the R-studio version 4.1.2 program. Biological control was calculated using the following formula:

(1) $$B.C.=\left(\frac{Individuals\;afected\;by\;the\;natural\;enemy}{Healthy\;Individuals}\right)\times 100$$

**Foliar consumption rate.** In total, 43 larvae of the first instar, 36 of the second and third instar, 33 of the fourth, and 46 of the fifth instar were evaluated to quantify foliar consumption. All the larvae were reared separately in Petri dishes, and a 7 × 4 cm piece of leaflet was taken from leaf 17 of a Compacta × Ghana cultivar palm. The leaflet was changed every three days, and the leaf area consumed was calculated. For this, photos were taken before and after the leaflet pieces were offered to the larvae and then analyzed with the Image J^®^ program developed by Wayne Rasband (Wisconsin, USA). The data were analyzed through descriptive statistics.

## 3. Results

**Figure 1:**
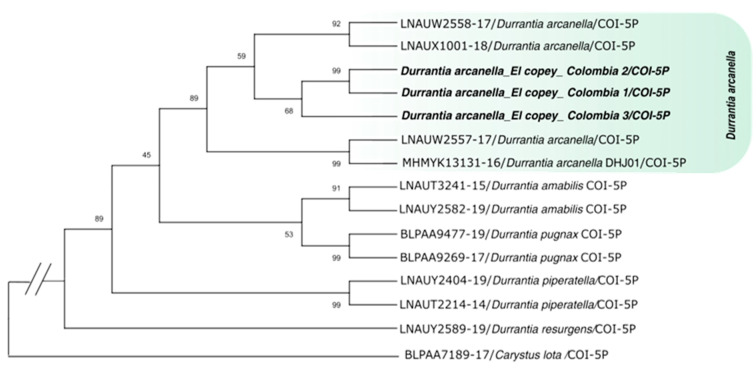
Phylogenetic tree obtained by the Maximum Likelihood statistical method based on the Cytochrome Oxidase 1 (COI) gene for *Durrantia* sp. species.

**Molecular identification and phylogenetic analysis.** Each of the *Durrantia* stages was molecularly identified with the COI gene, obtaining a percentage of similarity for the adult stage of 99.51% (Copey_Colombia 1), larva of 99.50% (Copey_Colombia 2), and pupa of 98.67% (Copey_Colombia 3) with the species *Durrantia arcanella*. The sequences of adult, larva, and pupa were related to sequences of samples collected and reported via Boldsystem from Panama (CCDB-29465-B04 and CCDB-32975-E03), Mexico (CCDB-29465-B03), and Costa Rica (09-SRNP-103185). According to the Maximum Likelihood phylogenetic tree (Figure 1), the *Durrantia* consensus sequences obtained in this study were grouped in the same clade with the reference sequences of the species *D. arcanella* recorded in Boldsystem (LNAUW2557-17, MHMYK13131-16, LNAUW2558-17, and LNAUX1001-18) with a higher bootstrap support of 89%. Likewise, *D. arcanella* was separated from other species of the same genus.

**Biology of *Durrantia* arcanella.** The total duration of the life cycle of *D*. *arcanella* was 48.0 ± 10.1 days under laboratory conditions (28.2 ± 2.5 °C, 82.1 ± 10% R.H.).

**Figure 2:**
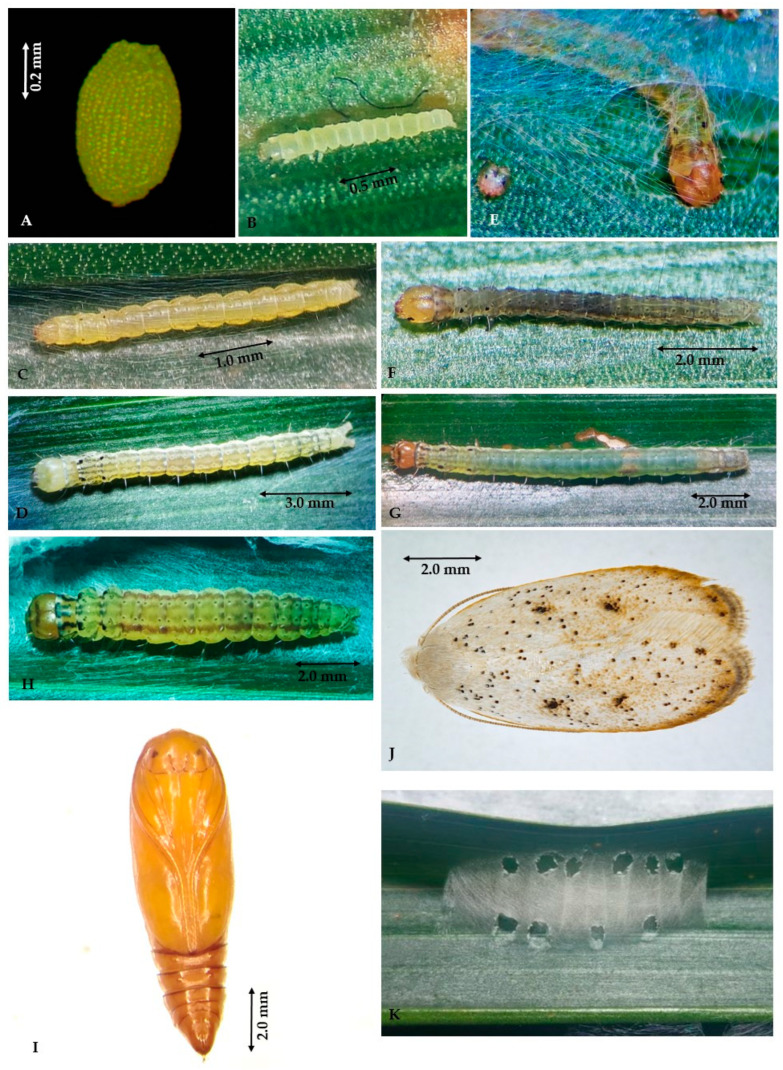
Developmental stages of Durrantia *arcanella*: (**A**) egg, (**B**) larva I, (**C**) larva II, (**D**) larva IV, (**E**) protective silk tissue constructed by the larva, (**F**) larva III, (**G**) larva V, (**H**) pre-pupa, (**I**) pupa, (**J**) adult, (**K**) reinforced silk tissue where the pupa develops.

**Eggs.** Initially, the eggs are shiny yellowish green in color, oval-shaped, measuring 0.5 ± 0.1 mm long and 0.3 ± 0.08 mm wide. The chorion comprises cells in irregular polygons with a slightly roughened texture (Figure 2A); after 3–4 days, the egg surface changes to a dull light-yellow color and simple eyes of larvae are perceivable. The completely developed larvae may be observed through the chorion on days 6–7. Hatching occurs in about 8.0 ± 0.7 days when neonate larvae move their mouthparts to make an aperture on the eggshell. They have a viability average of 70.4 ± 5.0%.

**Larvae.** The larvae go through five instars (Table 1); they have an elongated and cylindrical body of the eruciform type with the presence of simple setae, they have a sclerotized brown head of the hypognathous type with chewing mouthparts, stemmata situated at both epicranial regions formed by six lateral single-lens eyes, five arranged as a semicircle and one more located ventrally, with short antennae with three segments. They also present three pairs of well-developed thoracic legs, four pairs of pseudolegs located on the third, fourth, fifth, and sixth abdominal segments, and one pair of anal pseudolegs. When hatching, the first instar larvae are light-yellow and have an average length of 2.1 ± 0.5 mm (Figure 2B). Once they start feeding, they change to a light green and increase in size. The second instar larvae measure 3.3 ± 0.9 mm in length and are characterized by four black dots on the dorsal part of the mesothorax and metathorax (Figure 2C). After ecdysis, the instars III, IV, and V have a pale yellow body and head until they turn green over time (Figure 2D); they have solitary habits and take refuge under a white silk tissue that they build on the bundle or the underside of the leaflet (Figure 2E). They are characterized by a band of four irregular dark lines that start on the prothoracic shield and run longitudinally along the back until becoming opaque on the anal shield. At the end of their larval development, they measure 17.9 ± 2.5 mm in length (Figure 2G). At this point, they stop feeding and begin the pre-pupa stage, which is marked by a reduction in larva size and by the construction of a thicker line on the underside of the leaflets, woven with three reinforced layers at the ends intended for the protection of the pupa (Figure 2K). The pre-pupa measures 11.4 ± 1.0 mm in length and is yellow-orange. On the dorsal part of the abdomen, the four irregular bands turn reddish as the insect reduces in size until it becomes a chrysalis (Figure 2H).

**Pupae.** The pupae are of the obtect type; their average length is 6.6 ± 0.6 mm for males and 8.0 ± 0.5 mm for females; and at the beginning of their development, they are light orange, turning dark orange and finally brown (Figure 2I). A morphological identification feature for sex determination is visible on the ventral side of the abdomen: the female genital suture covers the eighth abdominal segment completely, whereas the male genital suture is positioned in the middle section of the ninth abdominal segment.

**Adult.** The adult has a body covered with mostly creamy white scales, simple developed maxillary palps, long and recurved labial palps, developed proboscis, and oval-shaped forewings with two dark brown spots in the middle of each wing and a line light brown that borders the forewing from the apical end and becomes thinner until ending at the costal margin (Figure 2J). There is also sexual dimorphism: The male has slightly serrated antennae and slender abdomen, measuring 6.6 ± 0.4 mm in length with a wingspan of 14.1 ± 1.1 mm. In contrast, the female has simple antennae and a wider abdomen, a length of 8.6 ± 0.5 mm with a wingspan of 17.8 ± 0.7 mm; the ovipositor is visible at the end of the last abdomen segment. The natural sex ratio is about two females for each male. Each female of *D*. *arcanella* oviposits an average of 143.2 ± 55.3 eggs during its adult stage.

**Figure 3:**
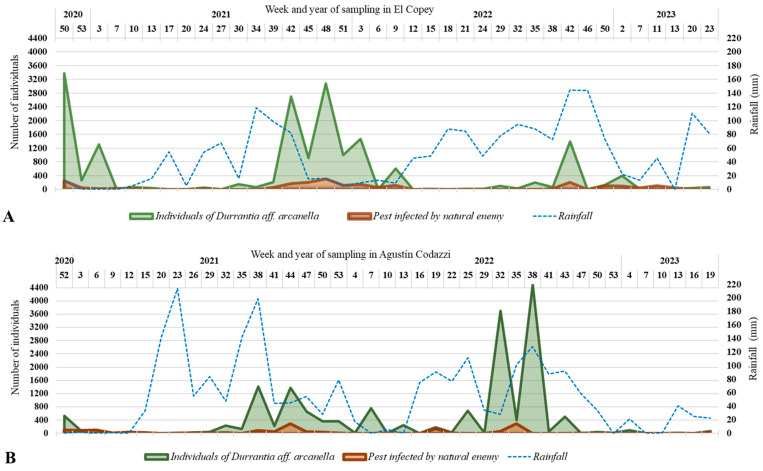
Population fluctuation in *Durrantia arcanella* and its relationship with natural control and precipitation in two oil palm plots: (**A**) In the municipality of El Copey. (**B**). In the municipality of Agustín Codazzi.

**Figure 4:**
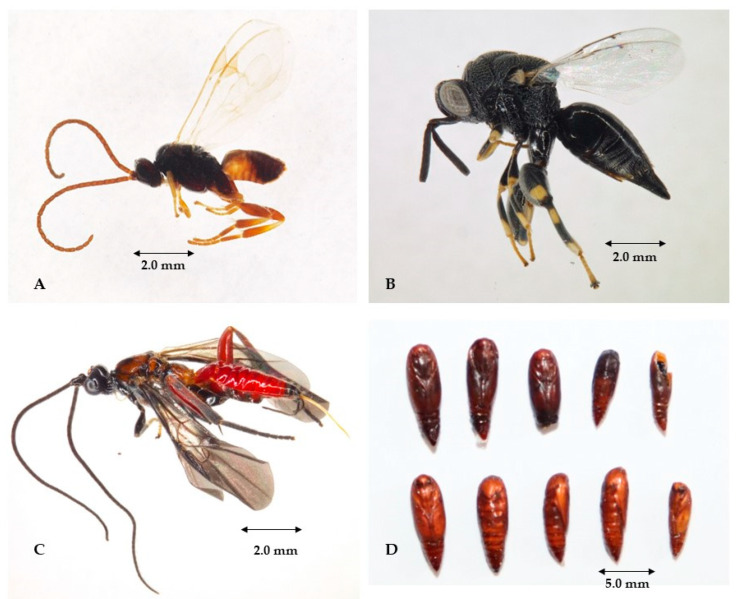
Natural enemies of *Durrantia*. *arcanella* in the department of Cesar: (**A**) *Gnamptodon* sp. (**B**) *Brachymeria* sp. (**C**) *Neorharcodes* sp. (**D**) Above: pupae parasitized by *Brachymeria* sp. Below: healthy pupae.

**Population fluctuation in *Durrantia arcanella*.** During the 30 months, 41 samples were taken, where a total of 15953 larvae and 2712 pupae were recorded in the plot located in El Copey and 21,190 larvae and 2855 pupae in the plot located in Agustín Codazzi. Each year in both locations, populations were below 800 individuals from February to July, and values above 1200 were recorded from August to January. The highest population peaks were observed in the plot in Agustín Codazzi in August–September 2022 (Figure 3A) and El Copey during December 2020 and September–October 2021 (Figure 3B). During the sampling in both plantations, the presence of natural enemies was recorded, which controlled 11.2% of the total individuals in the lot located in El Copey and 8.1% of those registered in Agustín Codazzi. The most frequent natural enemy was *Gnamptodon* sp. (Hymenoptera: Braconidae), a solitary endoparasitoid of first- and second-instar larvae of *D*. *arcanella* (Figure 4A), responsible for 40.3% of biological control, followed by *Brachymeria* sp. (Hymenoptera: Chalcididae) 29.1%, a solitary endoparasitoid of pupae (Figure 4B–D). The entomopathogenic fungus *Cordyceps* sp. (Hypocreales: Cordycipitaceae) was found to affect pupae, representing 24.9% of the natural control. Finally, *Neorharcodes* sp. (Hymenoptera: Ichneumonidae) pupal parasitoid 5.6% (Figure 4C). 

There was no association between population density and precipitation; however (Table 2), in El Copey, negative correlations were recorded between the population dynamics of *D. arcanella* larvae and pupae and temperature (R = −0.4500; *p* < 0.0043), positive correlations were recorded between population dynamics and biological control (R = 0.6100; *p* < 0.000044), and negative correlations were recorded between temperature and biological control (R = −0.6300; *p* < 0.000024). Meanwhile, in Agustín Codazzi, the population had positive correlations with relative humidity (R = 0.3300; *p* < 0.0340) and with biological control (R = 0.4200; *p* < 0.0060).

**Figure 5:**
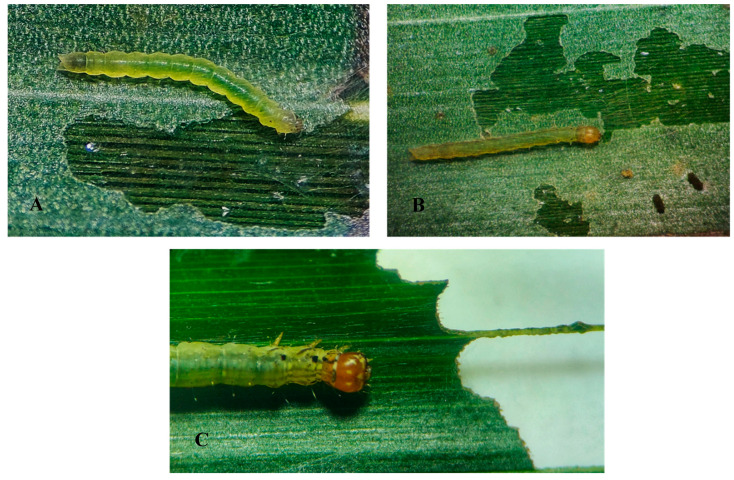
(**A**) Larva of the second instar of *Durrantia arcanella* causing gnawing on the leaflet. (**B**) Larva of third instar. (**C**) Fourth instar of *D. arcanella* completely consuming the leaflet.

**Foliar consumption rate.** The first- to third-instar larvae cause scratches on the underside of the leaf blade on the leaves of levels 9 and 17 of the oil palm (Figure 5A,B); the fourth- and fifth-instar larvae can completely ingest the leaf blade, mainly on levels 17, 25, and 33 (Figure 4C). Each individual completes its larval development after consuming 8.23 ± 5.3 cm^2^ of leaf blade (Table 3).

## 4. Discussion

The information obtained on the duration of the life cycle of *D. arcanella*, of 48.0 ± 10.1 days and with five larval instars, differs from that recorded by other authors; [11] indicates a total duration of the life cycle of 75 days; meanwhile, ref. [9] recorded that this insect goes through four larval instars with a duration of 34 to 43 days. These authors recorded the population parameters through field observations. However, they did not describe the environmental conditions in which the experiment was carried out, making a more precise comparison of the results difficult. Relative to other insects in the family Depressariidae, the life cycle of *D. arcanella* was similar to that recorded for *Cerconota anonella* Sepp., 1830, under laboratory conditions [22] and much lower than that reported for *Opisina arenosella* Walker, 1864, (75 days) in coconut and *Struthocelis semiotarsa* Meyrick cultures (82 to 92 days) in oil palm [11,23].

The morphological description of the different stages of development of *D. arcanella* coincides with the characteristics reported in the two studies on the biology of this species. Other investigations that also reported five larval instars in Depressariidae species also mentioned how the refuge strategies of the larvae are one of the leading evolutionary characteristics of this family [3,7,9,11,22,23,24].

The study of the population fluctuation in *D*. *arcanella* in the two localities of the department of Cesar showed similar cyclical population patterns in the same periods each year. This pattern could be linked to the insect’s life cycle and, among other things, to climate factors and agronomic practices [25]. However, no association was found between population dynamics and precipitation, but a positive correlation with relative humidity was recorded in Agustín Codazzi and a negative correlation with temperature in El Copey; this suggests that even when the populations of *D. arcanella* did not present a direct relationship with precipitation, it was observed that approximately 20 weeks after the start of the wet period the number of larvae increased in both locations, indicating that the population thrives in periods with higher humidity and lower average temperatures. These conditions allow a generational increase and higher survival rate of the population progeny after progeny until reaching high infestations in the second half of the year, bringing negative implications for the crop. Other studies have already stated that temperature is one of the climatic factors that affect the dynamics of phytophagous insect populations, directly impacting the survival, development rates, voltinism, longevity, fecundity, and oviposition of females [5,12,25,26,27,28,29,30,31,32].

Biological control provided by the natural enemies of *D. arcanella* presented a strong positive correlation with the population of the pest insect and a negative correlation with the temperature; this agrees with studies that report that these biological control agents respond quickly to changes in the population dynamics of their host and to climatic variations [30,33]. Additionally, parasitoid insects from the Braconidae, Chalcididae, and Ichneumonidae families have been reported as important biological controllers not only in the Depressariidae family but also in other Lepidoptera [22,34,35,36,37,38].

The foliar consumption rate estimated in this study shows that, individually, a larva of *D. arcanella* can consume up to 8.23 cm^2^ of the leaf blade; this value is much lower compared to other Lepidoptera that are also a pest of oil palm such as *Opsiphanes cassina* (425.5 cm^2^), *Euprosterna elaeasa* (66 cm^2^), *Stenoma cecropia* (36.7 cm^2^), and *Sibine megasomoides* (1427 cm^2^) [5,12,13,39]. Despite this, the short life cycle compared to these defoliating insects of *Elaeis guineensis* allows *D. arcanella* to be a multivoltine species in oil palm and produce 7–8 generations per year; this, together with the increase in Pestalotiopsis, can generate defoliation that could cause severe economic losses if timely detection and control measures are not taken to counteract the effects that this insect could cause on the crop. However, it is necessary to determine action thresholds to quantify the economic damage to which the crop could be exposed [4,10,15,40].

## Figures and Tables

**Table 1:** Duration in days (± standard deviation) of the different stages of *Durrantia arcanella* in oil palm (Compacta × Ghana) leaflets under laboratory conditions (28.2 ± 2.5 °C, 82 ± 10% RH).

Estadio	*n*	Average ± S.D. (Days)	Min.–Max. (Days)
Egg	389	8.0 ± 0.7	7.0–9.0
Larva I instar	87	4.4 ± 1.2	3.0–7.0
Larva II instar	55	4.3 ± 1.3	3.0–6.0
Larva III instar	55	5.6 ± 1.4	3.0–7.0
Larva IV instar	49	5.0 ± 1.4	3.4–7.0
Larva V instar	47	4.9 ± 1.2	4.0–6.0
Pre-pupa	45	1.5 ± 0.5	1.0–2.4
Pupa	94	7.1 ± 0.9	5.0–9.3
Adult	94	7.2 ± 2.0	4.0–12.0
**Total life cycle**		**48.0 ± 10.1**	**33.4–65.7**

**Table 2:** Spearman’s correlation relating the population dynamics of *D*. * arcanella* with some climatic variables and biological control in 2 oil palm plantations.

Localization		Precipitation	Relative Humidity	Temperature	Biological Control
**El Copey**	*D.* * arcanella*	−0.0770	0.1700	−0.4500	0.6100
	***p***	0.6500	0.3000	0.0043 **	4.4 × 10^−5^ **
	Biological Control	−0.1900	0.1200	−0.6300	
	***p***	0.2400	0.4600	2.4 × 10^−5^ **	
**Codazzi**	*D.* * arcanella*	0.2100	0.3300	−0.2100	0.4200
	***p***	0.1800	0.0340 **	0.1900	0.006 **
	Biological Control	0.0270	0.1500	0.1100	
	***p***	0.8700	0.3600	0.5000	

** Significance with α = 0.01.

**Table 3:** The foliar consumption rate of the immature stages of *Durrantia arcanella* under laboratory conditions (28.2 ± 2.5 °C, 82 ± 10% RH).

Larval Instar	Mean ± S.D. (cm^2^)	Interval Min.–Max. (cm^2^)
Instar I	0.151 ± 0.06	0.09–0.47
Instar II	0.29 ± 0.10	0.17–0.53
Instar III	1.22 ± 1.28	0.50–5.75
Instar IV	2.44 ± 1.98	0.90–8.60
Instar V	4.13 ± 1.87	2.05–9.66
Total	8.23 ± 5.30	3.71–24.98

## Data Availability

Data supporting this research are available to the corresponding author upon request.
